# Psychedelics and autobiographical memory – six open questions

**DOI:** 10.1007/s00213-025-06771-5

**Published:** 2025-03-17

**Authors:** Samuli Kangaslampi, Morten Lietz

**Affiliations:** 1 Faculty of Social Sciences / Psychology, Tampere University, Tampere, Finland; 2 Department of Neuropsychology and Psychopharmacology, Faculty of Psychology and Neuroscience, Maastricht University, Maastricht, The Netherlands; 3 University Center for Psychiatric Research, University of Fribourg, Villars-sur-Glâne, Switzerland

**Keywords:** Psychedelics, Autobiographical memory, LSD, Psilocybin

## Abstract

**Rationale:**

Since the earliest LSD research, psychedelics have been claimed to enhance autobiographical memory. Revisiting and processing autobiographical memories has further been suggested to be a major component of the therapeutic action of psychedelics. However, modern psychedelic research has largely neglected autobiographical elements of psychedelic experiences, and many vital questions remain unanswered.

**Objectives:**

We present and discuss six open questions related to psychedelics and autobiographical memory: (1) Do psychedelics enhance autobiographical recall? (2) Is recall and processing of significant autobiographical (e.g., traumatic) memories a common part of psychedelic experiences? (3) Do psychedelics promote the development of false or inaccurate memories? (4) How do autobiographical memories change if they are recalled and reconsolidated under the effects of psychedelics? (5) What are memories of psychedelic experiences like? (6) Are autobiographical experiences under psychedelics of particular importance for their therapeutic effects?

**Results:**

We present the background and current limited state of evidence for each question and provide suggestions on how future studies could best address them.

**Conclusions:**

Besides advancing basic research, answering these pressing questions is highly relevant for the possible therapeutic use of psychedelics, both in terms of developing and optimizing new interventions and for avoiding iatrogenic harms. Ideally, future psychedelic-assisted interventions could harness the possible synergies between the effects of psychedelics and existing memory-based therapies.

## Introduction

Autobiographical memory is a form of self-referential memory that includes episodic memories and semantic knowledge of oneself (Conway and Pleydell-Pearce 2000). Episodic memories are hippocampal-dependent recollections involving mental time travel to specific events in one’s past, capturing details about where and when those events occurred. Semantic memory, in contrast, is cortical-dependent general knowledge about oneself, such as knowing where one was born without recalling the specific event. Autobiographical memory is an essential component of the human experience crucial to the sense of self and identity (e.g., McAdams 2008). Autobiographical memory recall can be assessed across domains of, e.g., accuracy, vividness, emotional intensity, specificity, richness of detail, and ease of access. Different mental health disorders show distinct alterations in these domains. For instance, major depressive disorder is associated with over-general and negatively biased memories (Hallford et al. 2021; Weiss-Cowie et al. 2023). Schizophrenia involves reduced specificity, richness of detail, and conscious recollection (Berna et al. 2016). In posttraumatic stress disorder (PTSD), over-general memory is again observed (Sumner 2012; Williams et al. 2007), traumatic memories are overly accessible and may be distorted, while vividness of non-traumatic memories is reduced (Schönfeld and Ehlers 2017) and memory processing is altered and negatively biased (Durand et al. 2019; Otgaar et al. 2017). Accordingly, the effects of any therapeutic intervention on autobiographical memory would be highly relevant for these disorders.

Already Busch and Johnson (1950) noted that classic psychedelics such as LSD allowed patients to revisit and vividly relive significant past experiences. Other early researchers also found that LSD facilitated vivid recollections of past events (Chandler and Hartman 1960; Cohen 1967; Leuner 1962; Sandison 1954, 1959; Stoll 1947). Sandison (1954) listed reliving of childhood memories as one of three major types of LSD experiences in his patients. Other cartographers of psychedelic experiences concurred (Grof 1975; Pahnke 1969; Richards 2008), naming such memory experiences *psychodynamic*, reflecting their psychoanalytic backgrounds. Reliving autobiographical memories was seen as a core therapeutic mechanism of classic psychedelics, as they were claimed to provide unique access to them.

In contrast, modern psychedelic research is seemingly less interested in autobiographical elements of psychedelic experiences. Instead, research has focused on mystical union, ego dissolution, breakthroughs, and other largely “non-personal” types of subjective experiences under psychedelics. Hence, we lack insight into psychedelics’ effects on autobiographical memory. It appears worth investigating whether the therapeutic effects of psychedelics might be directly or indirectly related to autobiographical memory processes, such as improvements in overgeneralized memory in depression or enhanced processing of traumatic memories in PTSD. In our view, a re-engagement with the memory-related effects of classic psychedelics is overdue. Besides possible acute effects on retrieval of previously encoded memories, these may include interesting effects on encoding new memories under their influence, or on consolidation of memories (changes in previously encoded memories). We list some pressing questions of high relevance in this area in Table 1, and discuss them below.

**Table 1:** Psychedelics and autobiographical memory – Six open questions

1. Do psychedelics enhance autobiographical recall?	
2. Is recall and processing of significant autobiographical (e.g., traumatic) memories a common part of psychedelic experiences?	
3. Do psychedelics promote the development of false or inaccurate memories?	
4. How do autobiographical memories change if they are recalled and reconsolidated under the effects of psychedelics?	
5. What are memories of psychedelic experiences like?	
6. Are autobiographical experiences under psychedelics of particular importance for their therapeutic effects?	

### Do psychedelics enhance autobiographical recall?

Early authors emphasized the unique ability of LSD to allow the reliving of memories with particular vividness, intensity, or realism, even akin to age regression (Leuner 1962; Sandison 1959). Claims were further made that participants had enhanced access to early childhood memories or repressed or forgotten memories (e.g., Busch and Johnson 1950; Chandler and Hartmann 1960; Cohen 1967; Grof 1975; Leuner 1962; Sandison 1959) - effects that today’s memory research would consider unlikely, if not impossible (Otgaar et al. 2021).

In more recent qualitative research, people do also report vivid autobiographical experiences under psychedelics (Gasser et al. 2015; Gonzáles et al. 2019; Horák et al. 2018; Watts et al. 2017). For example, Gasser et al. (2015, p. 9) note that some patients in their trial of LSD-assisted psychotherapy for anxiety related to life-threatening illness experienced “(sometimes hypermnestic) reliving of incidents from the past”.

However, very few studies have attempted to look at different domains of autobiographical memory recall in more precise or quantitative terms. As relates to enhancement of the subjective experience of memory recall, in one small study, psilocybin increased subjective phenomenal intensity of positive autobiographical memories (vividness, visual imagery, emotional intensity, and positive valence) (Carhart-Harris et al. 2012). Such effects may not be limited to autobiographical experiences, though, as similar enhancement of vividness and emotionality was observed for LSD in a guided mental imagery task (Kraehenmann et al. 2017).

As regards more objective measures, psychedelics dose-dependently impair performance in typical memory tasks involving encoding of non-autobiographical material under their influence (Doss et al. 2024b; Healy 2021). With respect to memory retrieval, so far we have no empirical evidence that psychedelics would acutely enhance autobiographical recall in the sense of more accurate or detailed memories or better access to memories. LSD has in fact been found to reduce mental time travel to the past (Speth et al. 2016), and temporary amnesia of the personal past and identity are anecdotally reported under psychedelics (Milliére et al. 2018). Dose may well play a role: As effects like ego dissolution are more pronounced at higher doses (Holze et al. 2020), lower doses like those used in the psycholytic approach (30–150 µg of LSD or 3‒15 mg of psilocybin; see Passie et al. 2022), might be better suited to enhance (at least subjective experience of) autobiographical memory recall. Additionally, external factors such as setting, context, and expectations likely influence the extent to which autobiographical memory is accessed. Future studies could test these hypotheses by varying doses and adapting the setting to encourage autobiographical experiences using, e.g., cueing paradigms, a pre-prepared autobiography (MacLean et al. 1961), photos or other material, or specific instructions.

Still, determining whether psychedelics truly enhance autobiographical memory in the sense of more accurate recall or better access to memories remains challenging due to the subjective nature of memory, particularly given psychedelics’ effects on salience, significance, and sense of certainty (Carhart-Harris et al. 2012; Yaden et al. 2017). Any subjectively reported improvement in autobiographical memory under psychedelics may well reflect changes in perception, experience, and confidence rather than true gains in accuracy or access. Some options do exist, however. For example, diary methods where participants record true, altered, and false events daily for several weeks before psychedelic (vs. placebo) ingestion could provide one option to study effects on autobiographical memory retrieval, including on accuracy and access, conscious recollection vs. simply knowing, and subjective phenomenology at recall versus at time of encoding (Pernot-Marino et al. 2004; Conway 1996).

### Is recall and processing of significant autobiographical (e.g., traumatic) memories a common part of psychedelic experiences?

While anecdotal and historical reports suggest that psychedelics often bring up past memories, including difficult or traumatic ones, there is surprisingly little quantitative data on what types of memories people typically recall during these experiences. One recent study found that recollection of past adverse life events was common in ayahuasca ceremonies (Weiss et al. 2023), but more research is needed across different contexts and substances. Understanding whether the recall of autobiographical memories, especially those that are traumatic or emotionally intense, is a frequent part of psychedelic experiences is crucial. If these substances frequently precipitate the recall and emotional processing of such memories, this further raises the question of whether this is inherently therapeutic or whether it may, in some or most cases, cause harm or distress.

Since psychedelics generally heighten emotionality and amplify access to a wide range of emotions, both positive and negative, they may profoundly influence retrieval of emotional autobiographical memories. Psychedelics are known to induce a positive affective bias in mood, behavior, and emotion recognition (Griffiths et al. 2006; Kometer et al. 2012; Kraehenmann et al. 2015; Mallaroni et al. 2023), which may increase the retrieval of emotionally significant positive memories. At the same time, their ability to intensify emotional access may facilitate emotional breakthroughs, moments of cathartic processing of difficult emotions that have been strongly associated with clinical improvements (Roseman et al. 2019). For example, a study on bereavement found that emotional breakthroughs during psychedelic experiences, likely stemming from autobiographical memories associated with the loss, were significantly linked to reductions in grief symptoms (Low and Earleywine 2024). Understanding the conditions under which positive and negative autobiographical memories are recalled and processed under psychedelics could help inform the further development of safe and effective settings for psychedelic therapy.

### Do psychedelics promote the development of false or inaccurate memories?

While psychedelics may increase or intensify the recall of (emotional) memories, a critical concern is their potential to distort memory accuracy. Early LSD research already acknowledged that psychedelic experiences often involve a mix of actual memories, fantasies, symbolic portrayals, and fragmented recollections (Chandler and Hartman 1960; Cohen 1967; Leuner 1962; Pahnke 1969; Sandison 1954). Reports of recalling infancy, birth, and even past lives (Grof 1975; Sandison 1959) further suggest that at least some experiences of first-person recall of past events under psychedelics do not in fact represent retrieval of veridical memories. Indeed, psychedelics may influence memory encoding and retrieval in ways that increase susceptibility to false memories.

Research by Doss et al. (2024a, b) suggests that psychedelics may distort episodic familiarity, so that familiarity is later misattributed to stimuli that are similar to those that were encoded under psychedelics, relevant for possible false memory formation. Memory distortion is not unique to classic psychedelics; other psychoactive substances like cannabis, sedatives, and ketamine have been shown to disrupt memory retrieval and increase false memories (Kloft et al. 2020; Doss et al. 2023). Notably, MDMA can impair hippocampal-dependent recollection while enhancing cortical-dependent familiarity, leading to false recognition of novel but related stimuli (Kloft et al. 2022). Classic psychedelics, meanwhile, appear to heighten suggestibility and amplify perceptions of salience, significance, and certainty (Carhart-Harris et al. 2012, 2015; Davis et al. 2020; Doss, Samaha et al. 2024; Hartogsohn 2018; Yaden et al. 2017). Together these effects raise concerns about creating or reinforcing inaccurate memories, particularly in clinical settings.

In terms of memory retrieval, recent qualitative studies report cases of apparent traumatic memories emerging during psychedelic therapy that had not been previously recalled, leading to uncertainty about their veracity (Aixalá 2022; Noorani et al. 2018; Nordin et al. 2024; Watts et al. 2017). With psychedelic-related adverse events increasingly being discussed (Breeksema et al. 2022; Evans et al. 2023), this potential highlights the need for systematic evaluation of prevalence rates and impact of such phenomena in patients and users. These concerns may also re-ignite debates over the nature of recovered and iatrogenic false memories more generally (McNally 2023; Otgaar et al. 2021).

### How do autobiographical memories change if they are recalled and reconsolidated under the effects of psychedelics?

Whenever a memory is retrieved, it may enter a period of reconsolidation, during which it is labile to change and updating (Schiller et al. 2022). Psychedelics may further enhance such lability and affect processes of de- and reconsolidation (Brouwer and Carhart-Harris 2021; Benvenuti et al. 2023; Doss et al. 2023), although direct evidence in humans is lacking. During reconsolidation, new information may become included in or associated with the activated memories (Hupbach et al. 2007). Given enhanced mental imagery, personal significance, sense of realness, and (positive) emotionality under psychedelics (Carhart-Harris et al. 2012; Davis et al. 2020; Griffiths et al. 2006; Kraehenmann et al. 2017; Yaden et al. 2017), memories recalled and reconsolidated under psychedelics could hypothetically become more vivid or emotional, more positive, more imbued with meaning or significance, or more strongly believed in in the longer term. However, we have no systematic experimental research on such changes, nor on whether or when they may be associated with therapeutic or negative long-term effects. This could well depend on the type of memories in question. For example, increased vividness of positive, life-affirming memories may be a boon, but increased vividness of negative memories could be harmful, though some evidence from rodent studies suggests increased vividness of negative memories may actually facilitate extinction learning (Struyf et al. 2018; Werle and Bertigo 2024). Increased belief could also be problematic for some memories, but useful for others. Understanding how recall and processing of different types of memories under psychedelics changes them could be crucial for refining approaches to psychedelic-assisted therapy. It could also reveal points of concern or potential sources of harm that need addressing.

### What are memories of psychedelic experiences like?

Already Klüver (1928) noted that “experiences in the mescal[ine] state are not easily forgotten.” Many people evaluate their psychedelic experiences as highly significant, memorable events (Davis et al. 2020; Griffiths et al. 2018; Nayak et al. 2023). In the long term, whatever significance and meaning these experiences continue to have in people’s lives will necessarily be mediated by the memory they have of them. A small pilot study recently suggested that pharmacologically blocking memory of the psychedelic experience may blunt the (positive) long-term effects of psilocybin (Nicholas et al. 2024). On the other hand, recollection of episodic memories encoded under psychedelics appears to be impaired, though familiarity may be enhanced (Doss et al. 2024). Even if psychedelic experiences are memorable, they may not be particularly well (i.e., accurately) remembered. Still, little research has examined psychedelic experiences *as autobiographical memories*. We know very little about them and their qualities in comparison with other memories.

Much could be learned by applying tools and methods already in use for studying other types of autobiographical memories (e.g., Berntsen and Rubin 2006; Boyacioglu and Akfirat 2015; Singer et al. 2013; Sutin and Robins 2007) to examine the contents and phenomenological qualities, subjective experience, life story integration or relevance, centrality, belief, and other aspects of memories of psychedelic experiences. These methods could help uncover whether some qualities of such psychedelic memories are associated with long-term effects on well-being or mental health, or with the re-experiencing of drug effects, sometimes reported after psychedelic use (Evans et al. 2023; Halpern et al. 2018). A further fruitful avenue could be studying how memories of psychedelic experiences change over time, whether naturally or with explicit integration practices, and whether such changes are relevant for therapeutic effects.

### Are autobiographical experiences under psychedelics of particular importance for their therapeutic effects?

The apparent ability of psychedelics to enhance vivid recall and emotional processing of especially early memories is of central importance to the psycholytic therapy approach (Healy 2021; Leuner 1962; Passie 2024). More recently, several authors have suggested recall and processing of memories to be a major component of the therapeutic action of psychedelics (e.g., Gasser et al. 2015; Inserra 2018; Nutt et al. 2020). Supporting this, Weiss et al. (2023) found that recollection of past adverse life events during ayahuasca ceremonies was positively associated with therapeutic effects.

Integrating emotionally significant memories into a coherent narrative has been argued to be critical for emotional regulation, identity development, and psychological well-being (e.g., Conway and Pleydell-Pearce 2000). Autobiographical memory integration, in particular, plays a central role in therapeutic processes, fostering personal growth and resilience (Habermas and Bluck 2000). Given that disruptions in autobiographical memory—such as over-generalization, negative biases, or reduced vividness—are common across various mental health disorders like depression and PTSD (Hallford et al. 2021; Schönfeld and Ehlers 2017; Weiss-Cowie et al. 2023), it is plausible that psychedelic therapy may operate, at least in part, through autobiographical memory-related mechanisms.

While recent psychedelic research has prioritized other therapeutic pathways, such as mystical-type experiences (Kangaslampi 2023), the role of memory-related processes should not be overlooked. Acute experiences of emotionally significant autobiographical memories during the psychedelic state may catalyze therapeutic change. Importantly, these memories might continue to influence psychological processing long after the drug effects have subsided, particularly during integrative sessions where they are revisited and woven into a coherent narrative.

Moreover, if re-examining and processing pivotal life experiences are found to be central to the therapeutic effects of psychedelics, this opens up possibilities for combining psychedelics with memory-based therapies. Exposure therapy is commonly used for PTSD, and psychedelics might enhance extinction learning, for example through the increased vividness of negative memories (Werle and Bertigo, 2024). Enhanced memory processing could lead to more effective therapeutic approaches. Besides exposure therapies for PTSD, these could include reminiscence therapy or life review, particularly for late-life depression. These therapies, which involve revisiting and reflecting on significant life experiences to foster positive autobiographical memories, have been linked to reduced depression and improved quality of life in older adults (Bohlmeijer et al. 2003; Khan et al. 2022; Lin et al. 2024). Although for now this remains speculative, psychedelics could potentially have significant synergies with such memory-focused therapies, making this combination an especially enticing area for future research.

## Conclusions

We have pointed out six pertinent open questions related to psychedelics and autobiographical memory, listed in Table 1. Besides advancing basic research on autobiographical memory and psychedelics in general, answering these questions about the memory effects of psychedelics and the nature of autobiographical psychedelic experiences is highly relevant for the possible therapeutic use of psychedelics.

To address these questions, future research should first seek to confirm whether psychedelics indeed enhance autobiographical memory in some sense—be it through improved access or accuracy, or increased vividness, specificity, or emotional intensity. It is equally important to ensure that such possible effects are generally beneficial to avoid iatrogenic harms, and to determine what factors—such as dosage, setting, or individual differences—modulate these memory effects. Experimental studies that vary these parameters and carefully assess memory outcomes using rigorous, quantitative methods will be essential.

In a best-case scenario, the successful elucidation of these mechanisms could lead to the development of refined therapeutic approaches that harness the synergies between psychedelics and memory-based therapies. For instance, new, potent psychedelic-assisted, autobiographical memory-based interventions could be offered for conditions like depression and PTSD, as well as for populations such as older adults, where autobiographical memory plays a crucial role in well-being. Such research may also lead us to re-examine therapeutic approaches already developed where the memory-related experiences induced by psychedelics and their emotional processing are a central feature (Leuner 1962; Passie 2024).

Considering how often it has been suggested that psychedelics have dramatic effects on autobiographical memory, it is surprising how little attention this aspect of their effects has received in the recent resurgence of research. It is high time to change this!

## Data Availability

Not applicable.
